# Supplementary material for: Satisfaction Paradoxes in Health Behaviors: Contrasting Patterns across Weight, Physical Activity and Dietary Habits
Source: Nutrients. 2024 Jul 12;16(14):2246. doi: 10.3390/nu16142246 (PMC11279640; doi:10.3390/nu16142246)
Supplement: Supplementary file 1 [file nutrients-16-02246-s001.zip › nutrients-3074060-supplementary.pdf]

**Table S1.** (**With cutoff 8**): The distribution of satisfaction level for weight, physical activity, and eating behaviors among 1957 university students in Jazan, Saudi Arabia.

| Characteristics | Dissatisfied [#] n (%) | Satisfied n (%) |
|---|---|---|
| **BMI categories** [a] | | |
| Underweight | 240 (58.54) | 170 (41.46) |
| Normal weight | 511 (48.71) | 538 (51.29) |
| Overweight | 288 (79.56) | 74 (20.44) |
| Obese | 126 (92.65) | 10 (7.35) |
| **Physical activity (Weekly)** | | |
| No physical activity | 675 (94.14) | 42 (5.86) |
| <150 min | 553 (77.02) | 165 (22.98) |
| ≥150 min | 237 (45.40) | 285 (54.60) |
| **Eating behaviors** | | |
| Consumption of whole-grain products | | |
| Yes | 785 (73.71) | 280 (26.29) |
| No | 666 (74.66) | 226 (25.34) |
| Consuming 5+ servings of fruits/vegetables (daily) | | |
| Yes | 332 (64.72) | 181 (35.28) |
| No | 1119 (77.49) | 325 (22.51) |
| Choosing low-fat meats | | |
| Yes | 654 (69.72) | 284 (30.28) |
| No | 797 (78.21) | 222 (21.79) |
| Choosing low-fat products | | |
| Yes | 428 (63.79) | 243 (36.21) |
| No | 1023 (79.55) | 263 (20.45) |
| Avoiding foods high in sugar | | |
| Yes | 504 (66.93) | 249 (33.07) |
| No | 947 (78.65) | 257 (21.35) |

BMI: body mass index; n: Sample size; IQR, Interquartile range. [a] BMI was calculated using the standard formula of weight in kilograms divided by height in meters squared. [#] Satisfaction was defined by a score of 8 or higher on a 10-point scale, while dissatisfaction was a score below 8.

**Table S2. (With cutoff 7):** The distribution of satisfaction level for weight, physical activity, and eating behaviors among 1957 university students in Jazan, Saudi Arabia.

| Characteristics | Dissatisfied[#] n (%) | Satisfied n (%) |
|---|---|---|
| **BMI categories[a]** | | |
| Underweight | 211 (51.46) | 199 (48.54) |
| Normal weight | 351 (33.46) | 698 (66.54) |
| Overweight | 236 (65.19) | 126 (34.81) |
| Obese | 119 (87.50) | 17 (12.50) |
| **Physical activity (Weekly)** | | |
| No physical activity | 658 (91.77) | 59 (8.23) |
| <150 min | 434 (60.45) | 284 (39.55) |
| ≥150 min | 163 (31.23) | 359 (68.77) |
| **Eating behaviors** | | |
| Consumption of whole-grain products | | |
| Yes | 635 (59.62) | 430 (40,38) |
| No | 569 (63,79) | 323 (36,21) |
| Consuming 5+ servings of fruits/vegetables (daily) | | |
| Yes | 266 (51,85) | 247 (48,15) |
| No | 938 (64,96) | 506 (35,04) |
| Choosing low-fat meats | | |
| Yes | 511 (54,48) | 427 (45,52) |
| No | 693 (68,01) | 326 (31,99) |
| Choosing low-fat products | | |
| Yes | 342 (50,97) | 329 (49,03) |
| No | 862 (67,03) | 424 (32,97) |
| Avoiding foods high in sugar | | |
| Yes | 393 (52,19) | 360 (47,81) |
| No | 811 (67,36) | 393 (32,64) |

BMI: body mass index; n: Sample size; IQR, Interquartile range. [a] BMI was calculated using the standard formula of weight in kilograms divided by height in meters squared. [#] Satisfaction was defined by a score of 7 or higher on a 10-point scale, while dissatisfaction was a score below 7.

**Table S3. (With cutoff 6):** The distribution of satisfaction level for weight, physical activity, and eating behaviors among 1957 university students in Jazan, Saudi Arabia.

| Characteristics | Dissatisfied [#] n (%) | Satisfied n (%) |
|---|---|---|
| **BMI categories** [a] | | |
| Underweight | 174 (42.44) | 236 (57.56) |
| Normal weight | 268 (25.55) | 781 (74.45) |
| Overweight | 198 (54.70) | 164 (45.30) |
| Obese | 112 (82.35) | 24 (17.65) |
| **Physical activity (Weekly)** | | |
| No physical activity | 631 (88.01) | 86 (11.99) |
| <150 min | 321 (44.71) | 397 (55.29) |
| ≥150 min | 118 (22.61) | 404 (77.39) |
| **Eating behaviors** | | |
| Consumption of whole-grain products | | |
| Yes | 489 (45.92) | 576 (54.08) |
| No | 470 (52.69) | 422 (47.31) |
| Consuming 5+ servings of fruits/vegetables (daily) | | |
| Yes | 200 (38.99) | 313 (61.01) |
| No | 759 (52.56) | 685 (47.44) |
| Choosing low-fat meats | | |
| Yes | 384 (40.94) | 554 (59.06) |
| No | 575 (56.43) | 444 (43.57) |
| Choosing low-fat products | | |
| Yes | 258 (38.45) | 413 (61.55) |
| No | 701 (54.51) | 585 (45.49) |
| Avoiding foods high in sugar | | |
| Yes | 303 (40.24) | 450 (59.76) |
| No | 656 (54.49) | 548 (45.51) |

BMI: body mass index; n: Sample size; IQR, Interquartile range. [a] BMI was calculated using the standard formula of weight in kilograms divided by height in meters squared. [#] Satisfaction was defined by a score of 6 or higher on a 10-point scale, while dissatisfaction was a score below 6.
